# The Glyco-Code Molecular Template Instructively Guides Skeletal and Neural Development, Tissue Regeneration and Dynamic Repair Responses Following Trauma and Disease

**DOI:** 10.3390/ijms27198646

**Published:** 2026-09-27

**Authors:** James Melrose

**Affiliations:** 1 Raymond Purves Bone and Joint Research Laboratories, Institute of Bone and Joint Lab, Kolling Institute, Faculty of Medicine and Health, The University of Sydney and the Northern Sydney Local Health District, Sydney, NSW 2065, Australia; james.melrose@sydney.edu.au; 2 Graduate School of Biomedical Engineering, University of New South Wales, Sydney, NSW 2052, Australia

**Keywords:** glycosaminoglycans, tissue repair, extracellular matrix, nerve repair, synapse, repair biology

## Abstract

Glycosaminoglycans encode instructional information that cells can decipher to regulate tissue development, repair responses, regeneration and recovery of tissue function following trauma or disease. This information has been termed the glyco-code and is a road map for tissue development and repair. This review shows that a clearer understanding of the glyco-code will support improved strategies in repair biology, particularly in skeletal and neural repair, regeneration, and therapeutic biomaterial design. Emerging therapeutic approaches for peripheral nerve repair increasingly combine multimodal delivery, microenvironmental control, and biomimetic structural support; however, the electroconductive properties of glycosaminoglycans have not yet been fully incorporated into these strategies. This gap is clinically important because neurological disorders affect more than 40% of the global population, representing approximately three billion people across conditions such as stroke, migraine, Alzheimer’s disease, and diabetic neuropathy. Nanoparticle-engineered platforms and glyco-code-driven biomaterials may, therefore, provide future regenerative therapies that integrate glycosaminoglycan structure, charge, ligand-binding capacity, informational functions, and electroconductive control of resident cell populations to drive neural recovery. Multifunctional glycosaminoglycan interactions with a diverse range of binding proteins orchestrate tissue form and function in a diverse range of tissues in health and disease and have been widely used in tissue engineering protocols in repair biology. Tissue atlases, advanced glycomics, and molecular dynamics binding studies are now identifying roles for glycosaminoglycan binding proteins in therapeutic repair processes at the single-cell level, aiding in the identification of molecular targets for more effective recovery of tissue function. DynamicBind and SurfDock are deep learning methods that accurately demonstrate state-of-the-art performance in understanding the docking of potential therapeutic drugs with prospective targets, identifying cryptic pockets in unseen protein targets, and accelerating the development of small molecules for previously undruggable targets, potentially expanding the horizons of computational drug discovery with glycan structures.

## 1. Introduction

Glycosaminoglycans (GAGs), including chondroitin sulfate (CS), dermatan sulfate (DS), heparan sulfate (HS), keratan sulfate (KS), and hyaluronan (HA), are key components of proteoglycans (PGs) and extracellular matrices (ECMs) in functional structures that provide mechanosensory cues to cells. This provides cells with sensory signaling properties that equip them with the ability to sense and respond to changes in their microenvironment during tissue development and following trauma or disease processes, and the ability to orchestrate repair processes, maintain tissue homeostasis and ECM organization, and support optimal tissue function [1]. HA, the only non-sulfated GAG, supports tissue homeostasis, tissue compartmentalization, hydration, and cytoprotection, forming HA–aggrecan aggregates that, in cartilage, equip this tissue with its weight-bearing properties [2] and in perineuronal nets (PNNs) in the CNS support synaptic cognitive processes [3,4]. GAG-decorated PGs occur on cell surfaces, within cells, and in the ECM, where specific sulfation and epimerization patterns affecting GAG sequences enable tissue- and time-dependent interactions with bioactive regulatory molecules to maintain tissue homeostasis and tissue function [5]. Evidence from animal models, human genetic disorders, and tissue engineering underscores the essential roles that GAGs play in normal tissue function and repair responses in trauma and disease. The cell-surface glycocalyx GAGs have molecular recognition and informational transfer properties from a glyco-code that helps regulate cell behavior, tissue organization, and tissue function by controlling how cells sense and respond to molecular and mechanical cues. These properties of the glycocalyx evolved through natural selection processes over an extended period of the evolution of life, where the glycocalyx had to develop responsive, resilient properties that ensured cellular survival amid a deluge of powerful interactive cues [6]. The endothelial glycocalyx is a good example illustrating this principle through regulation of vascular function, with shear-mediated mechanosensory signal transduction regulating blood volume through vasoconstrictive effects mediated by luminal endothelial and intimal smooth muscle cells to regulate blood volume and blood pressure [7,8]. The glycocalyx also has protective barrier functions, and when it is damaged during trauma or disease, glycan fragments released into the systemic circulation have been used as biomarkers to monitor the extent of tissue damage in disease processes and the effectiveness of therapeutic tissue repair responses and recovery of tissue function [9,10,11,12]. Some of these matricryptic ECM fragments also have tissue-reparative, cell-instructive properties that are not expressed by the intact polymeric native matrix molecule [13,14,15,16,17]. Matricryptic fragments of ECM molecules have been applied in a number of tissue repair strategies; many of these beneficial tissue reparative effects are mediated by the GAG components of these fragments [15,17,18,19]. Fragments of the HSPG, such as agrin, show promise in cardiac repair and regeneration and are cardioprotective [20,21]. Further, they can address NMJ degeneration in muscle wasting diseases [22] and have been used in normal and diabetic skin wound repair strategies [23,24]. Perlecan domain V and LG3 fragments have beneficial effects in the repair of the BBB following ischemic stroke [17,25,26]. The current review first outlines the structural and physicochemical diversity of the major GAG classes. It then examines how these molecules contribute to cellular communication, neurosensory function, skeletal development, and repair biology. Finally, it identifies emerging opportunities for GAG-informed biomaterials, tissue atlases, spatio-temporal mapping of GAG-protein interactions at the single-cell level in diseased tissues, and the identification of potential therapeutic targets in tissue repair strategies.

Development of deep learning (DL) models (DynamicBind [27], SurfDock [28]) in molecular docking studies represents computational approaches to predict how proteins interact with small-molecule ligands, incorporating the dynamic nature of such interactions and rare transitions between biologically relevant conformational equilibrium states [29]. These represent state-of-the-art developments providing a greater understanding of the effectiveness of docking interactions with prospective therapeutic drugs. These procedures may provide insightful information on how cryptic glycan interactions at the cell surface affect control of cellular behavior.

### 1.1. The Sophistication and Functional Cell Instructive Capability of the Glyco-Code

The glyco-code is a complex communication system rivaling DNA/RNA and protein systems in terms of informational complexity. Glycans are far more structurally diverse than proteins or nucleic acids and are not directly encoded by the genome but are synthesized through template-independent biosynthetic enzymatic networks. Throughout evolution, glycomolecules faced survival challenges and had to adapt to environmental pressures, generating cell- and organism-specific dynamic glycan instructive patterns that provide survival properties to organisms [6,30]. Protein glycosylation extends the limits of the genetic code, producing an almost unlimited range of structures that control cell and tissue functions [31]. Cell-based glycan arrays, glyco-genomic profiling, and advanced analytical glycomic methods define the structural determinants of glycan-protein interactions in living cells [32,33]. These approaches can clarify how changes in glycan structure affect tissue properties in disease and trauma, supporting the identification of molecular targets and the development of therapeutic interventions [34].

The recent development of parallel reaction monitoring-based infrared matrix-assisted laser desorption electrospray ionization mass spectrometry (PRM-IR-MALDESI) has enabled spatial mapping of complex CS isomer distributions and GAG signatures in ischemic stroke [35]. This approach can identify disease-associated glycan structures with high precision and may help focus therapeutic intervention strategies on defined molecular targets. Changes in tissue components can now be linked more directly with degenerative tissue effects, aiding in the interpretation of structure–function relationships in disease.

### 1.2. Physico-Chemical Properties of GAGs

GAGs are polyionic ECM components with high negative fixed charge density. Their associated counterions support water retention, ion flux regulation, and osmoregulatory cell signaling; these properties allow GAGs to influence membrane depolarization and cellular activation, particularly in excitable cell types such as neurons [36]. The ability of GAGs to bind large volumes of water underpins tissue hydration and, in specialized tissues such as diarthrodial joints, contributes to weight bearing and boundary lubrication. In cartilage, HA–aggrecan macro-forms organize the ECM and provide hydrodynamic properties that enable resistance to compressive forces [2]. In the CNS/PNS, neurons and glial cells produce GAGs decorating PGs that regulate development, plasticity, neuroinflammation, and tissue homeostasis in health and disease [34]. Disruption of PGs and their GAG side chains following brain or spinal cord injury rapidly alters the endothelial ECM, compromising the neurovascular unit and blood-brain barrier integrity, synaptic organization, and PNNs [34]. These changes impair neuronal function, cognitive performance, learning, and memory. Injury-induced depolymerization of HA generates bioactive fragments that inhibit oligodendrocyte-mediated remyelination [37,38], promote inflammation, angiogenesis, and matrix degradation through induction of active MMPs by a range of cells [39].

GAGs also regulate ionic balance and mechanotransduction through electrostatic interactions with ions, receptors, and ECM components [1]. HS- and CSPGs modulate tissue mechanics, integrin signaling, and receptor tyrosine kinase activity, influencing vascular development, immune regulation, and neuronal function. In the brain, dense PNN assemblies containing HA and aggrecan compartmentalize CNS tissue, maintain hydration, and protect the neuron from oxidant and mechanical stresses [3]; phosphacan also interacts with tenascin-R, organizing the architecture of PNNs [40] and regulating mechanotransductive signaling, such as the Hippo YAP/TAZ pathway, which influences neuronal differentiation, activity, and survival [41,42,43,44,45]. HA-aggregates are major functional macromolecular components of PNNs; these are neuroprotective structures that contribute to synaptic plasticity and neuronal activity [4].

### 1.3. GAG Biodiversity and Functional Properties Conveyed to Tissues

#### 1.3.1. Cellular Control Mediated by GAGs in Cell–Cell and Cell–ECM Communication

Pericellular and interstitial molecular networks involving PGs provide cells with a mechanosensing system that allows them to sense and respond to perturbations in their cellular microenvironments [46]. This enables cells to alter their metabolic profiles and replace ECM components depleted by disease or trauma, supporting cell-mediated regulation of tissue homeostasis and maintenance of optimal tissue properties. GAG-mediated glyco-code signals therefore contribute to cell–cell and cell–matrix communication, tissue development, homeostasis, and repair. GAGs also provide regenerative cues that have been incorporated into tissue-engineering approaches, some of which have reached clinical application [36,47,48,49].

#### 1.3.2. GAGs and Wnt Proteins in Stem Cell Renewal, Development of Migratory Pluripotent Stem Cell Lineages, Tissue Development, and Repair Responses

Stem cells drive tissue development, renewal, and regeneration and are regulated by their local niche environment. Short-range Wnt signals from the niche promote stem cell self-renewal, whereas cells that exit the Wnt signaling niche through interactions with ECM components such as HA, perlecan, and laminin differentiate and acquire pluripotent, migratory properties that allow remote participation in tissue development and repair. Wnt proteins establish the spatial limits of the stem cell niche [50], while GAGs encourage stem cell differentiation as part of an integrated program of tissue renewal and regeneration [51]. Toluidine blue-stained PNS and CNS tissue specimens show widespread GAG distribution, particularly in regions known to contain neuroprogenitor stem cells, including the subventricular regions, granular cells of the dentate gyrus, and the neurovascular unit [52,53] (Figure 1).

GAGs are widely distributed in the brain and spinal cord and are prominent in cartilage rudiments, which serve as transient informational developmental templates for cognitive processes and tissue morphogenesis and endochondral ossification during axial and appendicular skeletogenesis [56]. Diarthrodial joint cartilage also contains permanent cartilage on the articulating joint surfaces, where chondrocytes do not undergo the proliferative and differentiative changes found in growth plate cartilage [57]. Surface stem cell niches in articular cartilage contain chondroprogenitor cells involved in joint development [58,59]. These cells produce PGs bearing CS sulfation motifs, including 3B3[−], 4C3 and 7D4, which are biomarkers of joint development, ECM remodeling and tissue morphogenesis [60,61].

### 1.4. The Biodiverse Structure and Function of Specific GAG Classes

Five GAG classes are defined by their repeating disaccharide units and their linkages to PG core proteins; these are shown schematically in Figure 2. HA is the simplest GAG, yet it has diverse functional properties discussed later. HS is the most structurally complex GAG and undergoes extensive modification, giving it broad functional diversity in tissues. CS contains transient sulfation motifs that appear during embryonic tissue development and ECM remodeling in repair processes but are generally absent from mature tissues. In cartilage, CS contributes to structural stability, and the CS sulfation motifs 3B3[−], 4C3, and 7D4 serve as developmental markers of tissue morphogenesis.

The structure of keratan sulfate (KS) is based on the poly-*N*-Acetyl-lactosamine-Galβ(1–4) GlcNAcβ(1–3) template [62,63]. The KS chain is arranged into regions that are not sulfated (lactosamine regions), monosulfated, where GlcN is 6-*O-sulfated,* and regions that are disulfated, where D-galactose and GlcN are both 6-*O*-sulfated [63,64]. Three forms of KS have been identified, KS-I (cornea), KS-II (cartilage), and KS-III (brain), based on differences in their protein linkage structures [62,64,65]. These can be *O*-linked via serine/threonine residues or N-linked to asparagine residues on PG core proteins. These KS isoforms were named after the tissues where they were first identified; however, their distributions are not exclusive to these tissues, and they occur in a diverse range of tissues.

In common with other GAGs, sulfation is an important functional determinant of KS [66,67,68,69]. Its sulfated regions are variable in size depending on age and the spatial activity of KS glycosyltransferases and KS sulfotransferases, which assemble KS in tissues through a non-template-driven process. This results in variable spatio-temporal distributions of KS in specific tissue regions, producing considerable size and charge heterogeneity, spatial diversity, and variable functional properties of tissues.

**Figure 2:**
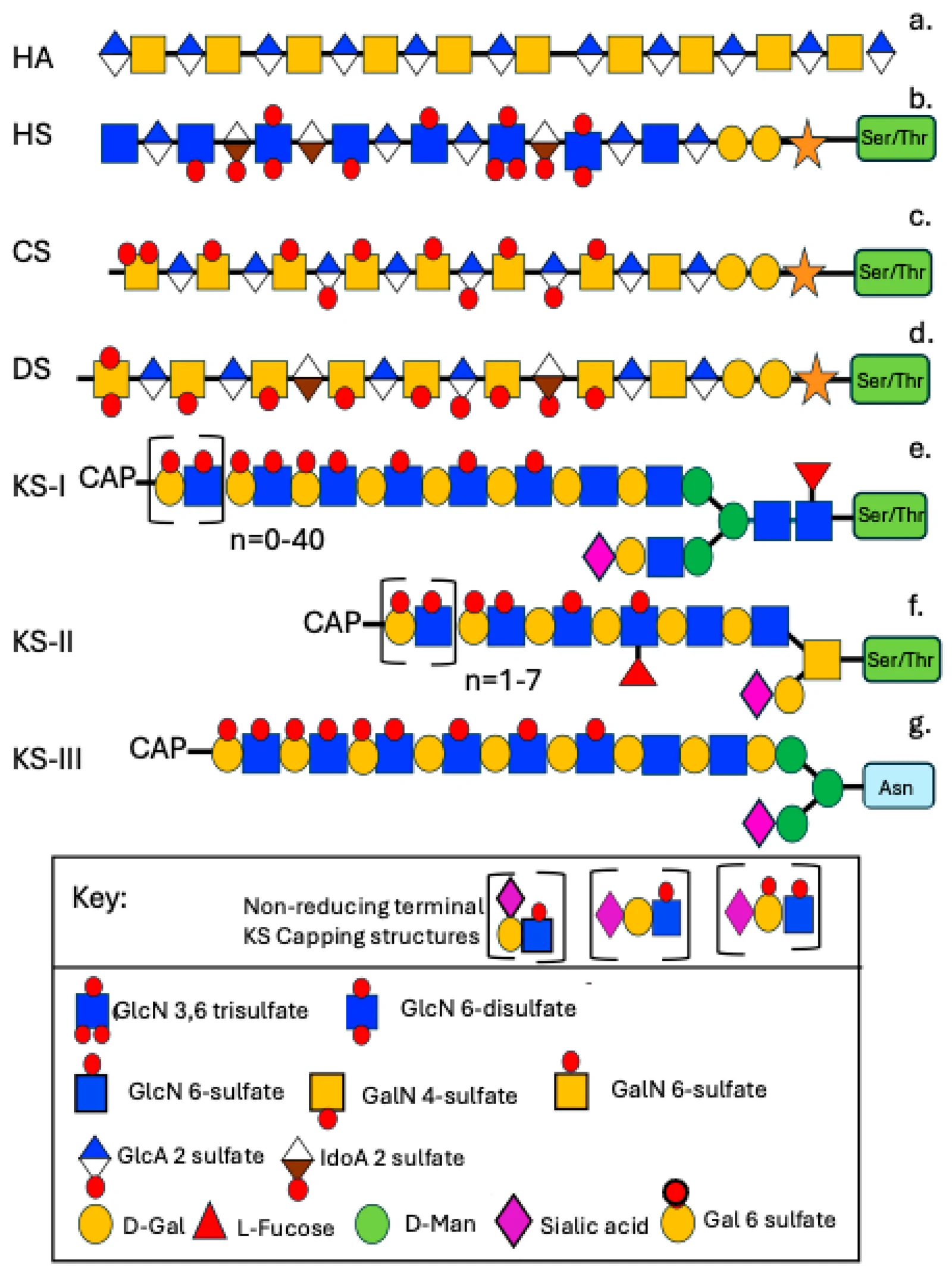
Schematic depictions of glycosaminoglycans hyaluronan (**a**), heparan sulfate (**b**), chondroitin sulfate (**c**), dermatan sulfate (**d**), corneal keratan sulfate (**e**), cartilage keratan sulfate (**f**), and brain keratan sulfate (**g**) showing their structural organization. The GAG chains shown are representative of each glycosaminoglycan class and illustrate their structural heterogeneity. KS has minor fucose and sialic acid capping structures on the non-reducing terminus, which increase resistance to enzymatic degradation. Proteomics studies show that corneal KS interacts with a diverse range of bioactive ligands [70]. The star symbol in HS and CS chains represents D-xylose a component of the linkage region attachment to serine or thronine residues in proteoglycan core proteins.

#### 1.4.1. HA, a Simple GAG but One with Diverse Functional Roles in Tissue Development

HA is a non-sulfated GAG that occurs as a 2–3 MDa polymer in synovial fluid and up to 7 MDa in some tumors. Polymeric HA is anti-inflammatory, scavenges ROS (reactive oxygen species), supports hydration, forms HA–aggrecan complexes that help cartilage resist compression, and supports neuronal cognitive processes in PNNs. As a major tissue ECM component, HA contributes to hydration, tissue elasticity, joint lubrication, and PNN plasticity [3]. HA localizes in tissues through interactions with HA receptors and selected PGs. Despite its simple structure, HA acts as an extracellular signaling platform through interactions with receptors and regulatory proteins [71,72]. It supports wound repair by promoting fibrin clot formation in early wound repair processes [73], maintaining a moist healing environment, and promoting fibroblast- and keratinocyte-mediated responses via CD44, EGF, and TGFβ receptor pathways. Polymeric HA is cytoprotective and anti-inflammatory, whereas depolymerized HA can promote inflammation, angiogenesis, and matrix remodeling, with potentially harmful effects in largely avascular load-bearing tissues. HA accumulates in demyelinated lesions, where hyaluronidase-derived oligomers support HA/Toll-like receptor-2 (TLR2) signaling and inhibit myelin repair by blocking late oligodendrocyte progenitor maturation [37,38,74]. Dysregulated HA metabolism is implicated in white matter injury, multiple sclerosis, and traumatic brain injury, with ~2 × 10^5^ Da HA fragments influencing oligodendrocyte precursor maturation and myelination/remyelination [37,38,75]. HA signaling regulates proliferation, differentiation, migration, and development in neural stem cells, neurons, astrocytes, microglia, and oligodendrocyte progenitors [71,72].

#### 1.4.2. The Complexity of HS Structure and Its Functional Interactive Motifs

HS is the most heterogeneous GAG after heparin. HS chains contain two main types of functional domains: (i) sulfated (S)-domains, enriched in highly modified disaccharides, i.e., sulfated hexuronic acid units linked to *O*-sulfated GlcNS residues, and (ii) *N*-acetylated (NA)-domains bearing mostly non-modified GlcNAc units linked predominantly to GlcA residues, although epimerization can also lead to Iduronic acid (IdoA). During its biosynthesis, HS undergoes modification, where unmodified GlcNAc residues linked to GlcA are targeted by N-Deacetylase/N-Sulfotransferases 1-4 (NDSTs 1-4), resulting in deacetylation prior to transfer of a sulfate group to the generated free amino groups, forming *N*-sulfated glucosamine (GlcNS). This is an important step essential to all remaining HS modifications, since most of the modifying enzymes act on GlcNS-containing motifs [76]. A small subset of *N*-deacetylated residues do not undergo *N*-sulfation, giving rise to unsubstituted glucosamine molecules (GlcNAc) [77], which are responsible for some highly specific interactions [78,79]. HS has diverse interactive properties with a range of bioactive molecules, binding to growth factors, chemokines, cytokines, morphogens, ECM proteins, enzymes, protease inhibitors, and transmembrane signaling receptors [5,80]. Several of the binding motifs for these bioactive molecules have been identified (Figure 3).

HS undergoes 2-*O*, 3-*O*, 6-*O,* and *N*-sulfation, and these sites determine the interactivity of HS. A specific trisaccharide, IdoA2S–GlcNS6S–IdoA2S, occurs in FGF1 binding sites in HS chains; 6-*O*-sulfation is important for FGF1-HS interaction [81]. IdoA2S and GlcNS are important units for FGF2 binding; 6-*O*-sulfated residues are also required to bridge FGF2 and FGFR, promoting formation of FGF2-FGFR-HS ternary complexes; this promotes receptor activation [82,83,84]. High 6-*O*-sulfation in the HS chains of syndecan-2 [85] results in increased VEGFA^165^ binding to HS chains [86].

The major morphogens Hedgehog (Hh), BMP, and Wnt are dependent on HS sulfation for interaction with HS. Wnt activation by HS requires 6-*O*-sulfation and is also influenced by post-translational 6-*O*-desulfation catalyzed by the 6-*O*-endosulfatases Sulf-1 and Sulf-2 [87,88]. The Wnt binding site on HS has been identified using a Wnt blocking antibody [88]. The most critical components of BMP-2 HS-binding domains are GlcNS and GlcNH_3_ residues; 6-*O*- and 2-*O*-sulfation are also important binding components but to a lesser extent [89]. The most selective interactions in HS are elicited by regions that contain rare modifications, such as GlcNH_3_ and 3-*O*-sulfated GlcN residues [77,90]. Studies on the anticoagulant properties of heparin identified an AT pentasaccharide GlcNAc6S-GlcA-GlcNS3S6S-IdoA2S-GlcNS6S, where the central 3-*O*-sulfation motif was shown to be critical for AT inhibitory activity [91]; 3-*O*-sulfation also significantly enhances tau binding to cell surface HS, promoting its cellular uptake and the prion-like spread of tau pathology in AD [92]. HS is found in nearly all mammalian cells and tissues as HSPGs, appearing on cell membranes, as components of specialized synaptic PGs, and throughout the ECM. HS is multifunctional [93] and performs biological functions specific to each tissue [94]. The HS side chains of HSPGs provide tissue protection by regulating physiological processes and cellular behavior [95]. HS is essential for tissue development [96], regulating processes such as morphogen storage, gradient formation, and cell signaling through interactions with key proteins such as growth factors, morphogens, and structural ECM proteins [97].

**Figure 3:**
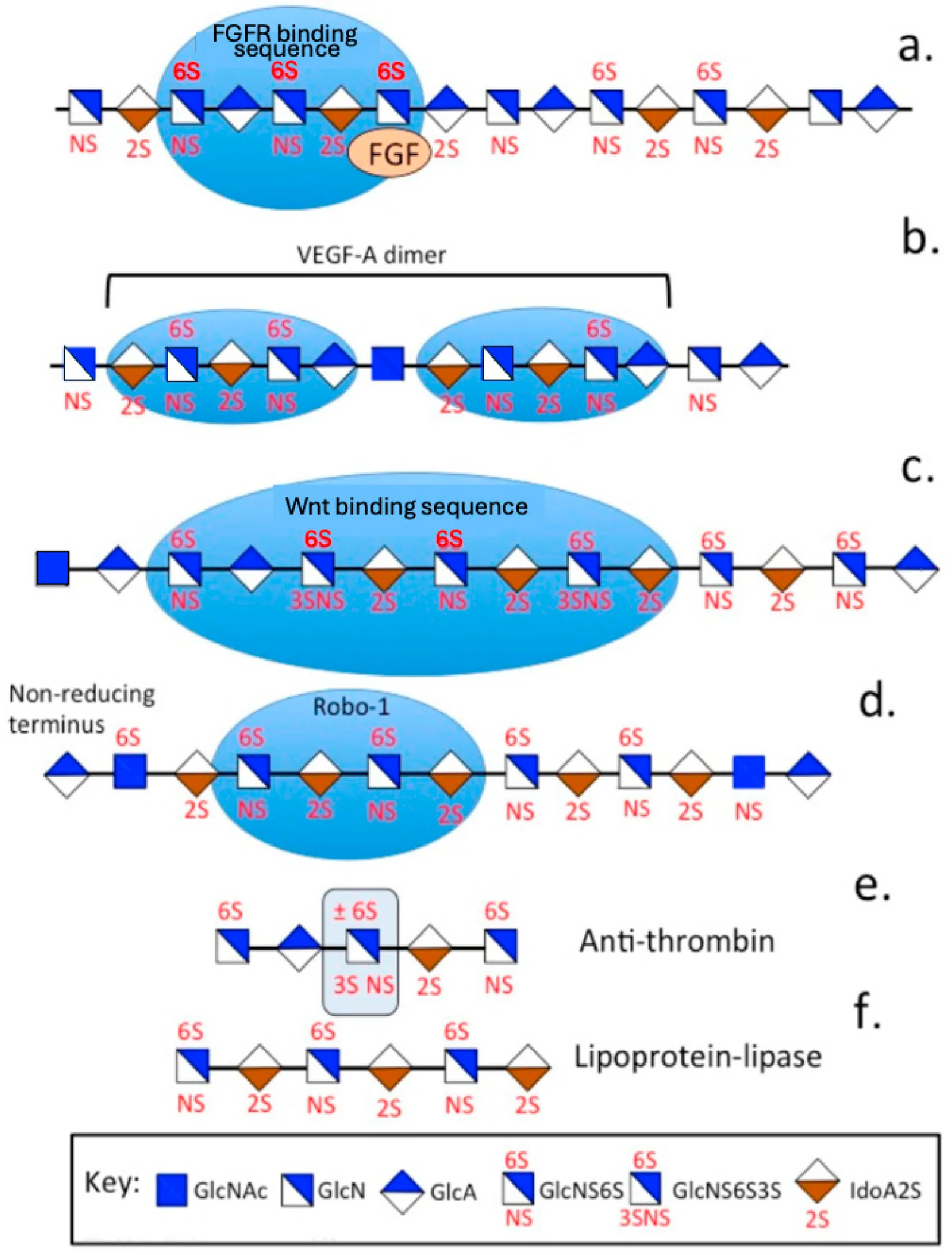
Several HS-binding proteins have been found to contain interactive HS sequences. FGFR and FGF-2 (**a**), VEGF-A dimer (**b**), Wnt binding protein sequence (**c**), Robo-1, roundabout receptor binding sequence (**d**), anti-thrombin pentasaccharide binding sequence (**e**), lipoprotein lipase binding sequence (**f**). Image reproduced from [95], accessed via Open Access.

HS is structurally related to heparin, which is a more highly sulfated variant restricted to mast cells, where it is attached to a central region of the core protein of serglycin, the smallest PG [98]. HS consists of repeating disaccharides of GlcNAc and GlcA, which undergo secondary modifications that generate structural diversity. Through its interactions with physiologically important proteins, HS plays a critical role in the development of ectodermal tissues, including the nervous system, eye, epidermis, bone, skin appendages, and tooth enamel [97]. The ability of HS to bind growth factors and present these to their cognate receptors in ternary complexes can promote receptor activation and proliferative effects on cells. HSPGs fine-tune mammalian physiology [99]. This has led to interest in HS as an agent that could potentially induce tissue regenerative effects in tissue engineering applications [100,101,102].

#### 1.4.3. Functional Roles for CS and DS in Tissue Development

CS and DS regulate tissue development, wound healing, and disease progression [103] and have specific functional roles in the nervous system [33] and in skeletogenesis [67,104]. As ECM scaffolding components, they support hydration, mechanical stability, and cell signaling, with important roles in neural tissue homeostasis and repair. CS and DS PG side chains contribute to growth factor and chemokine signaling and are essential in CNS development [33]. Disrupted DS biosynthesis causes connective tissue disorders, including spondylodysplastic Ehlers–Danlos syndrome, while DS-deficient mice show perinatal lethality, skin and vascular fragility, skeletal defects, myopathy, altered nerve regeneration, and impaired neural stem cell function.

Aggrecan, long considered a cartilage differentiation marker, is also expressed in the brain and notochord and is a key PNN component involved in cognition and synaptic plasticity [105]. Aggrecan components differentially modulate NGF-responsive and neurotrophin-3-responsive dorsal root ganglion neurite growth [106] and have neural crest cell-directive properties in embryonic development and neurogenesis [107]. Aggrecan CS chains guide axonal migration and neural network formation [108]. CS-composite scaffolds also direct development of chondroprogenitor stem cells [109], making CS attractive for biomaterial scaffolds that mimic native ECM environments and have been used to fabricate CS–HA composite osteochondral scaffolds [110].

CS sulfation motifs detected by the 3B3[−], 4C3, 7D4 and 6C3 MAbs identify tissues undergoing morphogenesis during development [60]. These motifs are not detectable in healthy mature tissues. However, when cartilaginous tissues undergo degeneration, these CS epitopes become detectable again [111]. While the precise structure of these CS sulfation structures has yet to be fully defined, differential digestion of CS chains using chondroitinases shows they have defined distributions along a CS chain. Initial digestion with chondroitinase ABC removes 6-C-3 immunoreactivity; continued digestion results in removal of 4-C-3 immunoreactivity and finally 7-D-4 immunoreactivity, indicating its location close to the CS-linkage region to the PG core protein [112]. The 3-B-3[−] epitope is an unsaturated non-reducing terminal epitope and should not be confused with the 3-B-3[+] epitope generated by chondroitinase digestion, which is located as a stub epitope close to the CS-linkage region. Hyaluronidase-4, which is a CS hydrolase despite its misnaming, is a mammalian enzyme produced by mast cells that also depolymerizes CS, generating the CS[−] epitope [113]. PGs carrying these epitopes are produced by chondroprogenitor stem cells during morphogenesis [114].

CS and DS-containing GalNAc4S6S were first identified in marine animals and later in humans. Their biosynthesis involves chondroitin 4-O-sulfotransferase-1 (C4ST-1), dermatan 4-O-sulfotransferase-1 (D4ST-1), and N-acetyl galactosamine 4-sulfate 6-O-sulfotransferase (GalNAc4S-6ST), whose products are implicated in CNS injury, cancer, fibrosis, osteoporosis, and viral infection. CS/DS GalNAc4S6S levels also increase during mast cell, macrophage, and neutrophil differentiation. Aggrecan in articular cartilage and brain PNNs derives ~90% of its mass from GAGs, which confer its polyionic hydration properties. In addition to abundant CS chains, aggrecan contains KS chains in the KS-rich region, G1 domain disulfide loops, and the interglobular domain (IGD) [2,115]. Some fucosylated KS chains occur in the CS1 and CS2 domains and are detected by antibody 3D12/H7 [116] (Figure 4). IGD KS chains are N-linked, less charged than other aggrecan KS chains, and may potentiate aggrecanase activity [115]. Brain aggrecan also contains human natural killer-1 (HNK-1) motifs (HSO3-3GlcA1-3Gal-1-NK-1ST) in CS1 and CS2 regions, replacing some CS chains, lowering negative charge density but adding ligand-interactive properties relevant to early notochord development, neural crest cell guidance, neuronal plasticity, and disease [105,117,118,119].

**Figure 4:**
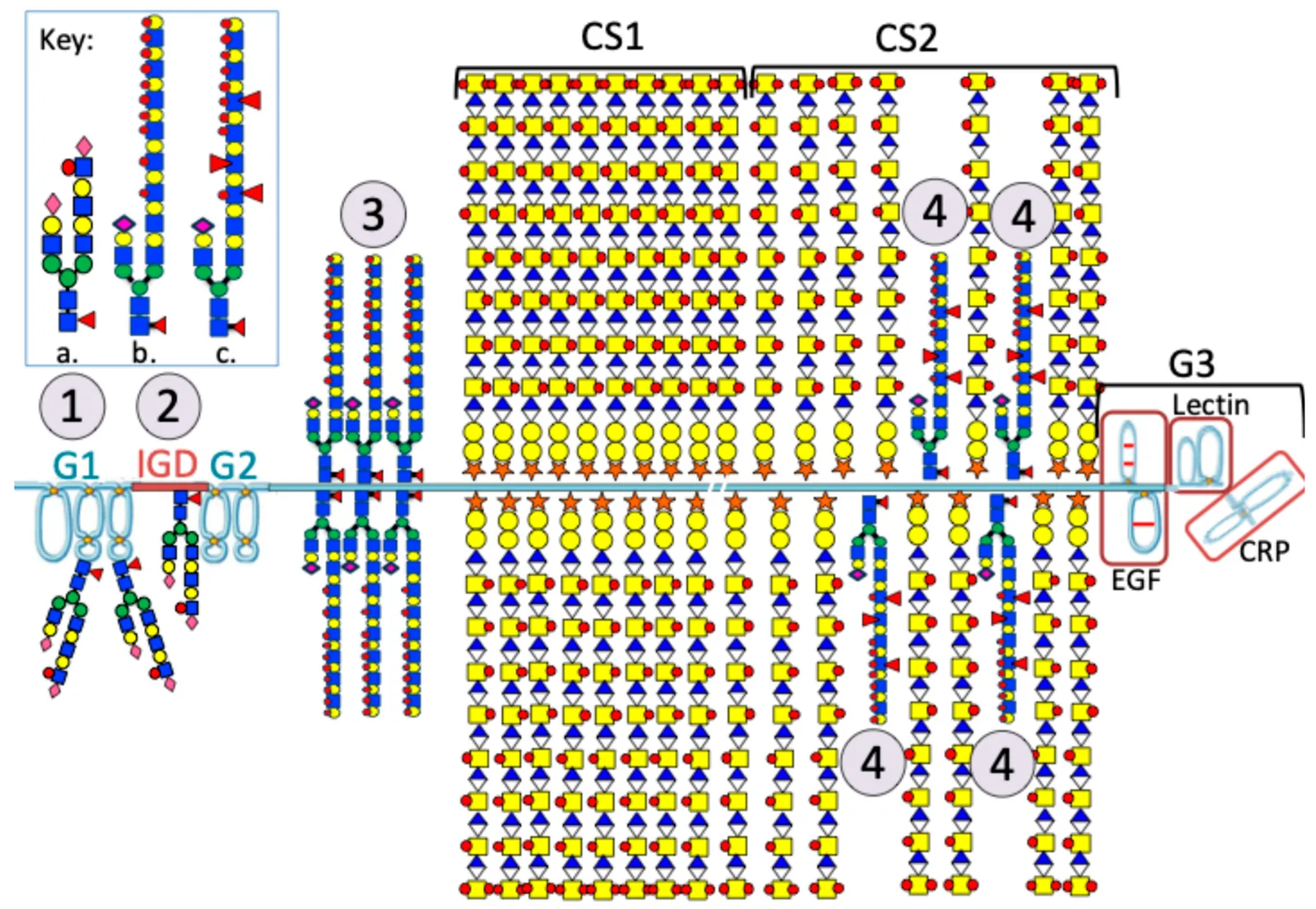
A schematic of a cartilage aggrecan molecule showing its G1 and G2 N-terminal and G3 C-terminal globular domains and its 250 kDa core protein, to which CS and KS chains are attached. G1 has HA-binding properties and aids in the formation of macromolecular aggregates in cartilage. CS is the major GAG in aggrecan; however, ACAN also has KS chains located in four regions along the core protein. The IgG folds in G1 (1) and IGD (2) contain small KS chains. KS chains in the KS-rich region (3) are approximately 7–10 kDa, similar in size to fucosylated KS chains within the CS1 and CS2 domains (4), which are detected using Mab 3D12/H7 [116]. All other KS chains are detected using Mab 5D4 except the IGD KS chains (a–c). The CS chains are around 20 kDa in size.

CS sulfation motifs detected by MAbs 3B3[−], 4C3, 6C3, and 7D4 are prominently expressed in developmental rudiment cartilage and intervertebral disc. Stem cells produce PGs bearing these CS sulfation motifs. During fetal knee joint development, 3B3[−], 7D4, and 4C3 motifs are widely expressed, especially in chondroprogenitor cells in cartilage surface regions [58,59]; 3B3[−] and 7D4 also mark developing fetal intervertebral disc regions, particularly the annulus fibrosus–vertebral body and inner annulus fibrosus–nucleus pulposus interfaces, the latter described as a developmental physis (Figure 5).

#### 1.4.4. KS Is a Highly Interactive Multifunctional Glycosaminoglycan

A proteomic and solid-phase binding study of corneal KS showed KS is a highly interactive GAG [62,70]. KS interacts with 85 kinases; kinases have diverse properties in the regulation of cellular physiological processes.

#### 1.4.5. Kinases Regulate Tissue Homeostasis

Interactions between KS and kinases are important for tissue function and the maintenance of tissue homeostasis in both health and disease [122,123]. Kinases are one of the largest and most diverse protein families in the human proteome, with more than 500 members, accounting for approximately 2% of the human genome [124,125].

#### 1.4.6. Phosphorylation as a Molecular Switch

Kinases regulate cell biology by transferring phosphate groups from high-energy molecules such as ATP to specific substrates, most commonly proteins. This reversible phosphorylation, together with dephosphorylation by phosphatases, modifies serine/threonine [126] and tyrosine residues [127,128], and can influence protein conformation and activation, subcellular localization, and protein–protein interactions [129,130]. Kinases act as molecular switches [131], transmitting signals that coordinate complex cellular processes.

#### 1.4.7. Physiological and Therapeutic Significance

Phosphorylation is central to regulating cell growth, metabolism, and apoptosis. Kinases are essential to diverse physiological processes in cancer, inflammatory disorders, and neurodegenerative diseases; thus, they are prominent therapeutic targets [132].

#### 1.4.8. Participation of KS-Proteoglycans in the Regulation of Tumor Development

##### Podocalyxin

Podocalyxin (PODXL) is a transmembrane PG negative regulator of human endometrial epithelial receptivity during embryo implantation. In the non-receptive state, PODXL helps maintain an endometrial surface that is less permissive to embryo attachment [133,134]. During the mid-secretory phase, PODXL is specifically downregulated in the luminal epithelium, aiding in the conversion of the endometrial surface to a state that permits embryo implantation, closely linked with the development of endometrial receptivity and important for successful embryo attachment and implantation.

Podocalyxin, a fucosylated, mucin-like sialomembrane proteoglycan related to CD34 [135], is a tumor-associated diagnostic and prognostic biomarker used to monitor chemoresistant cells [136]. Epithelial malignancies account for more than 90% of all human cancers. Cancer progression, driven by metastasis, immune evasion, and chemotherapy resistance, is associated with poor prognosis and adverse patient outcomes. PODXL is expressed in normal kidney podocytes, vascular endothelial cells, hematopoietic progenitor cells, mesothelium, and a subset of neurons. PODXL is an anti-adhesive epithelial cell molecule that regulates adhesion, cell morphology, kidney development, and renal function, contributing to tissue remodeling and development in many epithelial cancers, including lung [137], breast [138,139], colorectal [140], gastric [141], esophageal, and gastric adenocarcinoma [142], oral squamous cell carcinoma [143], mucinous ovarian carcinoma [144], papillary thyroid carcinoma [145], and epithelial serous and mucinous ovarian carcinoma [144]. PODXL is overexpressed in aggressive tumors and is associated with poor prognosis. PODXL-containing low-sulfated KS has been found in human testicular embryonal carcinoma, recognized by R-10G monoclonal antibody (Mab). Another low-sulfation KS antibody, 294-1B1, also showed by immunohistochemical analysis expression in embryonic carcinoma [146]. Double immunofluorescent staining showed colocalization of R-10G, 294-1B1, and PODXL immunoreactivity. Oversulfated KS is also highly expressed in glioblastoma and astrocytic tumors [147,148].

Among the general antibodies for human PODXL, TRA-1-60 and TRA-1-1 recognize its KS-related structures [135]. Mab 5-D-4 is a widely used anti-KS antibody [149] identifying a highly sulfated hexasaccharide KS epitope [150] in cornea, cartilaginous and non-cartilaginous tissues [151,152,153,154] in KSPGs including aggrecan, lumican, keratocan, fibromodulin [155,156]. A KS antibody, BKS-1[+], also detects a keratanase-generated neo-epitope in cartilaginous tissues [155].

##### Lumican

Lumican, a class II small leucine-rich proteoglycan (SLRP) substituted with up to four N-linked KS chains, has key roles in ECM organization and is a modulator of diverse biological functions [157]. Lumican is expressed in various cancer tissues and has a positive or negative correlation with tumor progression depending on tissue context [158,159,160,161]. The tumor microenvironment decisively controls cancer development by establishing a complex interplay between cancer cells and their stromal components, which directs disease progression [162,163]. Lumican can impede tumor cell migration and invasion through interactions with α2β1 integrin [164]. Lumican also has an MMP inhibitor module (lumcorin) located in LRR 9, which inhibits melanoma cell migration and also exerts angiostatic effects through α2β1 integrin and downregulates MMP-14 and MMP-9 associated with endothelial cell membranes; thus, lumican can display potent inhibitory activity against tumor progression [161,164]. Lumican is also involved in tumorigenic cellular processes such as epithelial-to-mesenchymal transition, cellular proliferation, migration, invasion, and adhesion in various cancer tissues [158].

Lumican inhibits MMP-14 and the attachment and proliferation of A375 and B16F1 melanoma tumor cells [165,166,167,168,169,170,171,172,173], inhibits pancreatic cancer cell growth [174,175,176,177], inhibits migration and invasion of lymph node, bone, and brain by metastatic prostate cancer cells [178], regulates osteosarcoma tumor cell adhesion by modulation of TGFβ2 activity, and is positively correlated with the differentiation and negatively with the growth of human osteosarcoma [179,180]. Cytoplasmic and rough endoplasmic reticulum (RER)-associated lumican in neuroendocrine tumors inhibits tumor growth [181]. Upregulation of lumican inhibits tumor cell migration and cellular proliferation in adenocarcinoma and squamous cell carcinoma of the lung [182,183].

#### 1.4.9. KS Is Electroconductive and Can Participate in Neurosensory Processes

KS is an electroconductive GAG that may participate in neuronal signaling through its ability to shuttle protons along the KS chain [184]. Emerging evidence suggests that KS may form part of a neuronal interface [185] that could enhance neuronal responsiveness. Proteomic analysis of corneal KS-I shows that it interacts with a diverse range of ligands, including neuroregulatory proteins [70], cytoskeletal proteins, receptors, growth factors, and kinases, supporting varied roles in CNS and PNS development. KS can trap and transfer protons through Grotthuss shuttling and may participate in neurotransmitter-related processes, making it an important functional GAG in neuronal tissues [186,187]. Proton shuttling or hopping has been proposed to represent a nerve conduction message [187]. As a multifunctional and electrosensory GAG, KS supports cell signaling and ECM organization, particularly in neural networks, and in some systems has roles in environmental sensing [188]. A low-sulfation KS isoform forms part of an ultrasensitive proton-detection gel in the Ampullae of Lorenzini of elasmobranch fish, enabling detection of extremely weak electric fields generated by prey fish species [186,189]. The American paddlefish even uses proton-mediated electrolocation to detect zooplankton, Daphnia, which it feeds on and that are beyond the limit of visual detection, and can detect these even in turbid water conditions [190]. Electrolocation mediated by KS-proton detection in sharks/rays represents an advanced biological sensory adaptation and the most sensitive proton-detection system so far identified in nature, with reported detection of electric fields as low as 5 nV/cm. Only two terrestrial animals have retained electrolocation as a sensory detection system; these are the duckbilled platypus and echidna monotreme animal species found in Australia [191,192]. However, while aquatic electrolocation no longer serves as a sensory system in the majority of vertebrates, this nevertheless shows the high sensory capabilities of KS. Further adaptation of the ultrasensitive properties of KS in sensory detection systems in mammals, potentially involving sensory hair-cell stereo-ciliary bundles, gelatinous membranes in the vestibular neurosensory epithelium, and cochlear temporal bones, suggests potential roles for KS in audition and the vestibular system that await full clarification [193,194,195,196].

## 2. Neurotransmitter Transport and Release During Neurotransmission

Neurotransmitters are stored in synaptic vesicles in the neuron soma. When a neuron is activated by an influx of Ca^2+^, the neuronal membrane depolarizes, and this depolarization travels down the axon to the synaptic gap (Figure 6). A transmembrane KSPG, SV2, is a smart storage and transport proteoglycan that transfers neurotransmitters entrapped in synaptic vesicles to the synaptic gap, where release of these neurotransmitters furthers neurotransmission processes. Synaptotagmin, a Ca^2+^ sensor protein, interacts with SV2 and aids in synchronized vesicle transport. Fusion of the synaptic vesicle with the depolarized presynaptic membrane releases neurotransmitters into the synaptic gap, where they are taken up by receptors on communicating dendrites on the postsynaptic membranes in the retinal network. This process is critical because dysfunctional neurotransmitter transmission is implicated in many brain disorders, including AD, PD, schizophrenia, depression, and drug addiction. Methods are available to monitor neurotransmitters and neuromodulators even up to the last millisecond of their life in a synaptic vesicle [197].

### 2.1. Stabilization of Synapses by Specialized HSPGs

Specialized HSPGs at synapses provide stability, specificity, and plasticity in neuronal communication (Figure 7). Neurexins regulate inhibitory and excitatory synapses through alternative splicing and HS modifications that variably affect the formation of complexes with synaptic proteins [198,199,200,201,202,203]. This forms a trans-synaptic code that shapes local neuronal circuitry, providing specificity in synaptic interactions and plasticity. Synaptic signaling is disrupted in neurodegenerative disease [204,205,206]. Pericellular PG networks help cells sense microenvironmental change, adjust metabolism, and restore ECM composition during development, disease, and repair. Pikachurin supports photoreceptor-bipolar neuron ribbon synapses essential for extremely rapid visual signaling [207], while Eyes-shut interacts with dystroglycan LamG domains (Figure 8) to stabilize the photoreceptor axoneme primary cilium, which attaches the inner and outer photoreceptor segments, stabilizing the photoreceptor [208,209]. HSPG-dystroglycan interactions through LamG domains stabilize synaptic connections and support synaptic plasticity and cell–ECM communication. The extensively glycosylated alpha-subunit of dystroglycan and matriglycan mediate these interactions, highlighting essential roles for GAGs in synaptic organization, neuronal communication, cell–matrix interaction, and tissue-specific functional regulation [36] (Figure 7 and Figure 8).

**Figure 7:**
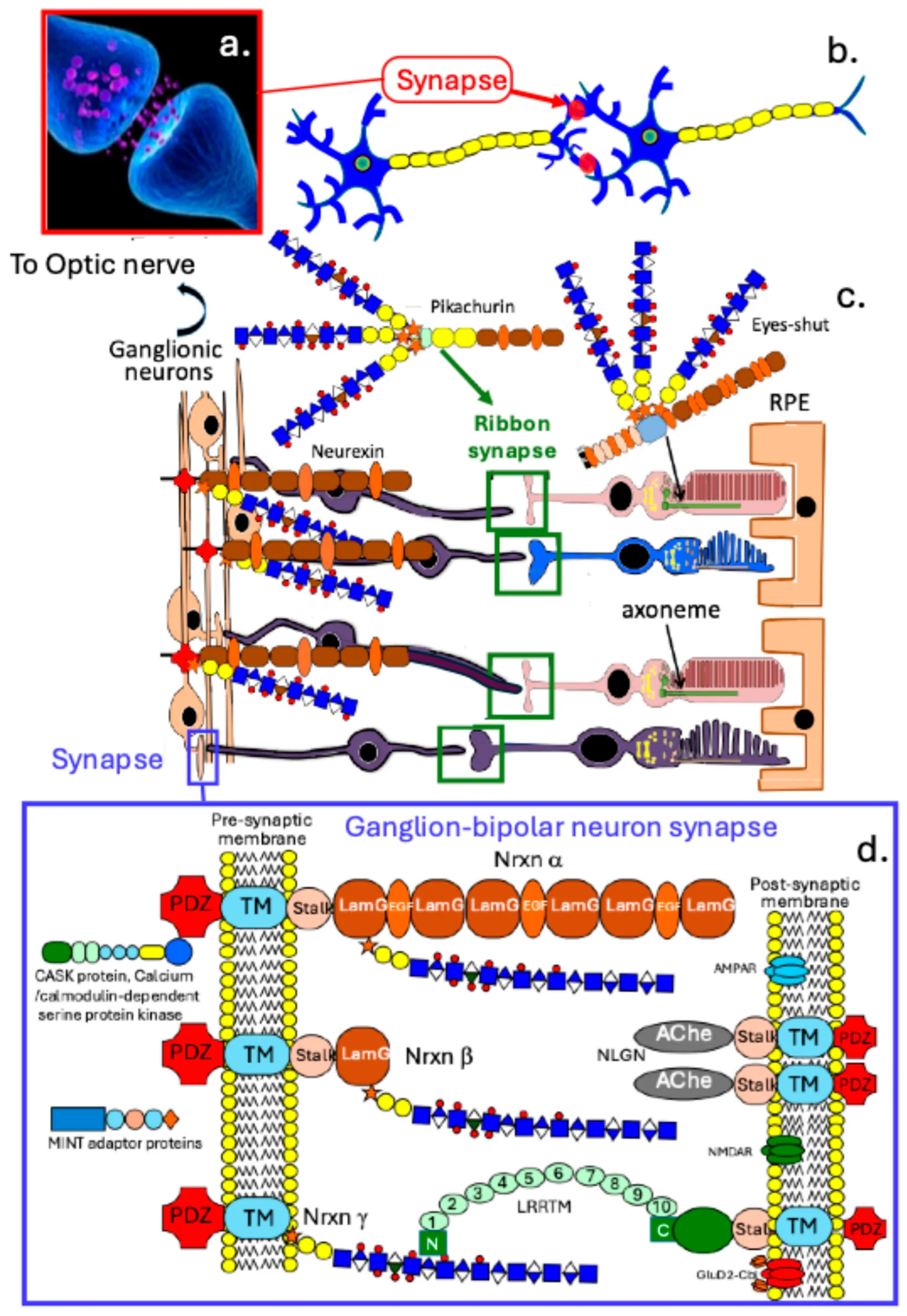
Schematic depictions of specialized HS-PGs, including pikachurin, Eyes-shut, and members of the neurexin family, showing their roles in synaptic stabilization, specificity, and plasticity. LamG and EGF domains are key interactive modules that interact with dystroglycan in these structures. Dystroglycan contains a heavily glycosylated alpha-subunit attached to a beta-subunit linked to cytoskeletal proteins and other components that facilitate cell–ECM communication (see Figure 8). Images modified from [184] and accessed via Open Access. (**a**) Artistic rendition of a synapse and a schematic of two interacting neurons (**b**). The retinal neural network mosaic shows interaction of retinal pigmented epithelial cells with photoreceptors, which interact with bipolar neurons and pikachurin in the ribbon synapse, while the neurexin family stabilizes ganglionic neuron synaptic interactions. (**d**) A key schematically depicts components represented in (**b**,**c**).

**Figure 8:**
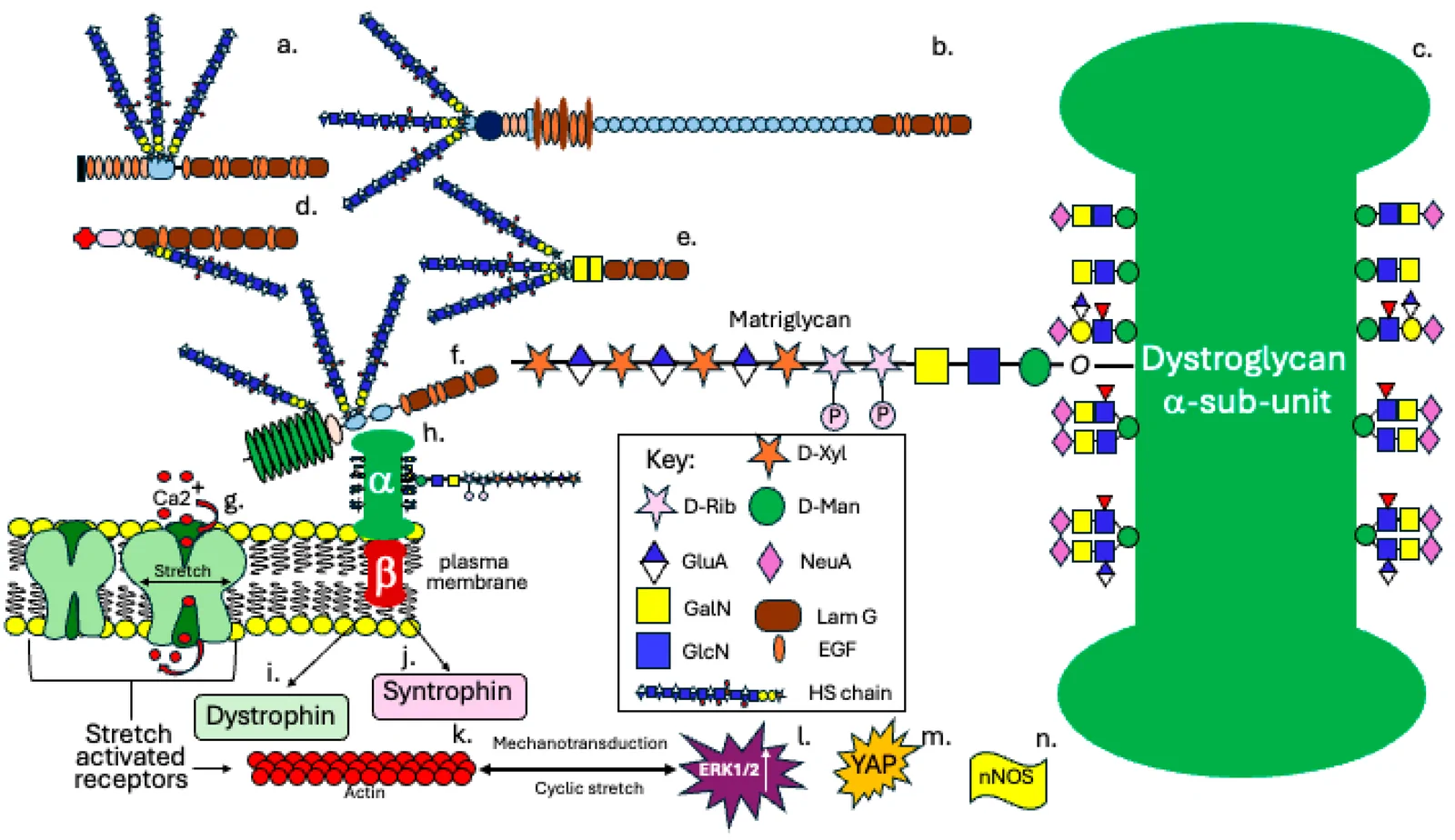
Schematic depiction of the dystroglycan interactive HS-proteoglycans Eyes-shut (**a**), perlecan (**b**), dystroglycan, including its extensive glycosylation (**c**), neurexin (**d**), pikachurin (**e**), agrin (**f**). Cell membrane dystroglycan interactive components include stretch activated receptors (**g**). The dystroglycan α, β subunits (**h**) form complexes with dystrophin (**i**) and syntrophin (**j**) regulating alterations in the actin skeleton (**k**) during mechanotransduction to regulate extracellular signal regulated kinase, ERK 1/2 (**l**), YAP (YES associated protein-1 transcription factor) (**m**) signaling and neuronal nitric oxide synthase (nNOS) activity (**n**). The key depicts dystroglycan glycan components interactive with ligands and laminin G and EGF modules in HS-proteoglycans interactive with the dystroglycan α-sub-unit. The beta-subunit of dystroglycan forms complexes with dynorphin, plectin, dystrobrevin, and syntrophin cytoskeletal proteins. Images modified from [208] using Open Access.

### 2.2. HSPGs Enhance Wnt and IHh Protein Solubility and Transport in Tissues

HSPGs regulate Wnt ligand dispersal, morphogen gradients, and cell-surface Wnt clustering in growth plate cartilage [210,211]. Through restricted diffusion involving repeated ligand binding and release, syndecans, glypicans, and related HSPGs control morphogen availability at the cell surface [212]. Perlecan LDLR domain II also binds and co-distributes Wnt and indian hedgehog (IHh) within growth plate gradients.

### 2.3. Binding of Indian Hedgehog by Perlecan

Perlecan binding of IHh regulates chondrocyte differentiation and proliferation during endochondral ossification. By directly binding IHh and modulating interactions with CSPGs such as aggrecan, perlecan helps coordinate bone formation and skeletal growth; this pathway is relevant to osteoarthritis and cartilaginous tumors with dysregulated IHh signaling [210,211]. Indian Hedgehog is regulated by Wnt signaling pathways in synovial joint formation and endochondral ossification [213,214]. Wnt/β-catenin and IHh pathways jointly regulate growth plate morphogenesis, endochondral ossification, osteoblast differentiation, chondrocyte proliferation, and hypertrophy [215,216,217,218,219]. Their interaction controls synovial joint formation and cartilage development, with IHh able to inhibit chondrocyte hypertrophy and joint formation independently of β-catenin, while Wnt9a spatially and temporally regulates IHh during chondrogenesis [217]. Wnt7b can substitute for IHh in inducing vascularization of hypertrophic cartilage but not osteoblast differentiation [218]. Proper CS sulfation is required for IHh binding to chondroitin-4-sulfate and aggrecan CS chains, highlighting the importance of GAG sulfation in growth plate morphogenesis and endochondral ossification [67,220]. IHh–Wnt pathway interactions are reciprocal and context-dependent during endochondral ossification. Wnt signaling can regulate IHh expression and activity, while IHh often suppresses Wnt/β-catenin signaling by promoting β-catenin degradation and destabilizing receptor complexes such as Frizzled and LRP5/6 [215,221]. These feedback loops fine-tune chondrocyte proliferation and hypertrophy, osteoblast differentiation, and joint formation, with additional regulation through chondroitin-4-sulfated aggrecan in the growth plate [67,104,220].

### 2.4. Roles for Wnt Gradients in Growth Plate Cartilage and Endochondral Ossification

Wnt signaling is essential for growth plate development and maintenance, regulating cell fate, proliferation, and tissue organization during endochondral ossification [216,222,223] (Figure 9). Proper Wnt activity establishes the chondrocyte columnar architecture required for bone growth, whereas dysregulation contributes to skeletal and stem cell disorders. Wnt proteins, including Wnt7a, have limited solubility but bind perlecan via its LDL receptor domain, enabling extracellular transport and gradient formation in skeletogenesis [223,224,225].

### 2.5. Wnt Signaling Orchestrates Cartilage Morphogenesis and Skeletal Development

Wnt signaling centrally regulates rudiment cartilage morphogenesis and skeletal development [223,226] (Table 1). Canonical and non-canonical Wnt pathways control chondrocyte differentiation, proliferation, and hypertrophy during cartilage formation and endochondral ossification [227]. Their dysregulation is linked to disorders such as osteoarthritis, supporting Wnt signaling as a therapeutic target [228].

### 2.6. BMPs and Cartilage

BMPs are TGF-β-related proteins that regulate chondrocyte and osteoblast development [229,230]. They bind perlecan mainly through HS and CS chains in domain I, where electrostatic interactions with sulfate and carboxyl groups create a protected local growth-factor pool for receptor presentation [231,232]. Perlecan increases BMP bioavailability and receptor activation during bone formation and repair, and recombinant perlecan domain I efficiently delivers BMP2 to promote osteoblast differentiation and bone formation [233]. Aggrecan CS chains provide an additional BMP reservoir in growth plate cartilage, while GAG-rich pericellular matrices concentrate growth factors near chondrocytes and support receptor clustering, ternary complex formation, and proliferative signaling [232].

**Table 1:** Wnt proteins involved in synovial joint formation, rudiment growth, cartilage development, growth plate cartilage morphogenesis, and endochondral ossification.

Developmental Stage	Wnt Protein Stimulation	Ref
Synovial joint cavitation	Wnt 14	[120,234]
Chondrogenesis; cell proliferation and differentiation	Wnt 9a, Wnt5a, Wnt 5b	[217,235,236]
Growth plate assembly Formation of chondrocyte columns	Wnt 5a	[235,237]
Hypertrophy, chondrocyte maturation Mineralization of cartilage	Wnt 4, Wnt 9a, Wnt 7b Wnt/β-catenin	[216,218,234,235,238,239,240,241]
Perichondrial bone formation Extension of axial skeleton	Wnt/β-catenin	[239,241]

## 3. Key Aspects of GAGs in the Regulation of Neural Tissues

GAGs regulate tissue development, homeostasis, and repair by instructing cells, directing ECM remodeling, and supporting wound healing after trauma, infection, or disease [242,243] and have considerable therapeutic potential [47], but their effects in neural tissues can be both beneficial and inhibitory. After CNS injury, CSPG accumulation in glial scars stabilizes damaged tissue but restricts axonal regeneration, particularly through inhibitory 4-sulfated GAG chains and altered expression of aggrecan, brevican, phosphacan, neurocan, and versican [244]. Aggrecan, HA, and aggrecan aggregates inhibit neurite outgrowth from dorsal root ganglion (DRG) neurons through CS chains, and chondroitinase digestion can reduce this inhibition; Rho kinase/ROCK (Rho-associated protein kinase) inhibition also enhances neurite growth in selected contexts [106]. Hyaluronidase-4 is a mammalian CS hydrolase rather than an HA-degrading enzyme, and has similar CS degradative properties to the bacterial chondroitinases, but its potential role in CNS repair remains to be established [245]. Because mast cells produce hyaluronidase-4 and operate during neuroinflammation, immune regulation may help overcome CS-mediated inhibition in glial scars [113]. More broadly, peripheral nerves regulate tissue repair [246] and electroconductive hydrogels can improve diabetic wound healing by modulating the neuro-immune microenvironment and promoting peripheral nerve regeneration [247]. Aggrecan also has beneficial roles in ECM-mediated neuroprotection and in PNNs, where it supports cognition and neural plasticity [248].

## 4. The Potential of GAGs in the Promotion of Wound Repair and Tissue Regeneration

Glycosaminoglycans and PGs promote wound healing and tissue regeneration [242,243,249,250]. Diabetic wounds heal poorly because of the hostile environment of these wounds due to immune dysregulation, neuropathy, infection, and chronic inflammation, which disrupt the neuro-immune wound environment. GAG-based therapies show promise in improving diabetic wound care and neural repair. Current strategies being investigated include GAG-mediated diabetic wound repair strategies [251], GAG-based macroporous scaffolds for growth-factor release [252,253], a number of nerve guidance conduits [254,255,256,257], electro-stimulation [258,259,260,261], and electric-eel-inspired ionogels [262,263]. An electroconductive anti-inflammatory chitosan/CS-A/hydroxyethyl cellulose ionogel conduit showed biocompatibility in a rat sciatic nerve model and promoted Schwann-cell activation, axonal guidance, remyelination, and functional recovery [264]. Other promising approaches in tissue repair include targeting senescent Schwann cells [265], injectable self-healing electroconductive HA-alginate hydrogels containing polypyrrole and the laminin-derived peptide IKVAV [266], advanced electroconductive hydrogels [267], multivalently engineered glyco-nanoplatforms for targeted therapy [268], controlled drug release from high-electroconductive-performance 3D printed scaffolds [269], extracellular vesicle delivery from skin-derived precursor Schwann cells [270] and use of Schwann cells for neural tissue repair [271,272,273].

## 5. Future GAG Research

Tissue atlases have advanced cell-level understanding of disease progression, therapeutic responses, and functional tissue organization at the single-cell level [274,275]. Spatial mapping of CS isomers and GAG signatures in ischemic stroke using PRM-IR-MALDESI mass spectrometry imaging has revealed disease-associated glycan structures and potential therapeutic targets [35]. More than 50% of proteins are known to undergo glycosylation, and growing evidence shows that glycosylation has pivotal roles in cellular differentiation, development, and disease [31]. A greater understanding of the cell-instructive cues delivered by GAG sequences and peptide modules may improve their use in biomedicine and therapeutic tissue-regeneration protocols in repair biology. GAGs are important information-storage and information-transfer molecules that provide cells with molecular recognition cues. Some peptides also display information-transfer and cell-instructive properties similar to those of GAGs. Extensive collaborative work by the TransCODE Consortium has produced protein-level evidence for non-canonical open reading frames (ncORFs), showing that approximately 25% of a set of 7264 ncORFs gave rise to detectable peptides in a large-scale analysis of 95,520 proteomics experiments [276]. Dark proteome ncORFs and their encoded miniproteins and peptideins identify a significant expansion of the information encoded by the human proteome. A peptidein is a small peptide molecule that mimics the actions of proteins. More than 1700 peptideins have been identified; most have unknown functions, but some are implicated in genetic diseases and cancer and have been referred to as dark proteins [277]. Miniproteins and peptideins may be useful in GAG pathobiology and repair biology studies and warrant future in-depth investigation.

## 6. Advances in Next-Generation Sequencing Illustrate the Importance of GAG Biosynthetic Enzymes in Tissue Function

The regulation of GAG biosynthesis remains incompletely understood; impaired sulfated GAG synthesis is increasingly linked to congenital connective tissue disorders [278,279]. Next-generation sequencing has identified causative genes affecting glycosyltransferases, sulfotransferases, isomerases, linker-region enzymes, and UDP-sugar or sulfate transporters [280,281,282]. Dysfunction in these pathways disrupts CS and HS synthesis, causing skeletal abnormalities and systemic effects involving the heart, kidneys, cognition, and immunity [278,283]. A greater understanding of glycosyltransferases, sulfotransferases, isomerases, and linker-region enzymes is thus warranted.

## 7. Conclusions

GAGs are bioregulatory molecules of considerable structural and functional complexity. This review has illustrated this through examples of structural effector molecules and the biological processes they regulate. These examples emphasize the information carried by GAGs, which evolved from the earliest stages of emerging life-forms and remain relevant in the present day in tissue development, mature tissue function, and cellular regulation. Despite the challenges of defining GAG structure–function relationships with binding proteins, new methodologies are improving understanding of these interactions and enabling increasingly effective applications in repair biology. Continued innovation in GAG-informed tissue repair and regeneration is therefore both necessary and promising. A summary of the valuable properties of GAGs is supplied below.

**Biological and Functional Advantages**

- Key regulators of cell behavior
  GAGs provide instructive biochemical signals (the “glyco code”) that regulate cell proliferation, migration, differentiation, and survival.
- Essential for tissue development and repair, orchestrating wound healing, ECM remodeling, and restoration of tissue homeostasis after injury.
- Structural and mechanical roles: High negative fixed charge density enables water retention, lubrication, shock absorption, and resistance to compression (e.g., cartilage and synovial joints).
- Growth factor modulation: GAGs bind, protect, and spatially present growth factors and morphogens (e.g., Wnt, BMP, FGF, Ihh), enhancing signaling precision.
- Neural protection and plasticity: In the nervous system, GAGs contribute to PNNs, synaptic stability, learning, memory, and controlled plasticity.
- Stem cell niche regulation: GAGs help define stem cell niches and guide lineage commitment and migration during development and repair.
- Therapeutic potential: GAGs have been successfully used or explored in the development of wound dressings (HA hydrogels), tissue engineering scaffolds (CS, HS, HA composites), and controlled growth factor delivery systems
- Electroconductive properties (notably KS): KS can conduct protons and participate in neurosensory and neuroregenerative processes, making it promising in nerve repair.
- High biocompatibility: As endogenous molecules, GAGs generally show low immunogenicity.

## Figures and Tables

**Figure 1:**
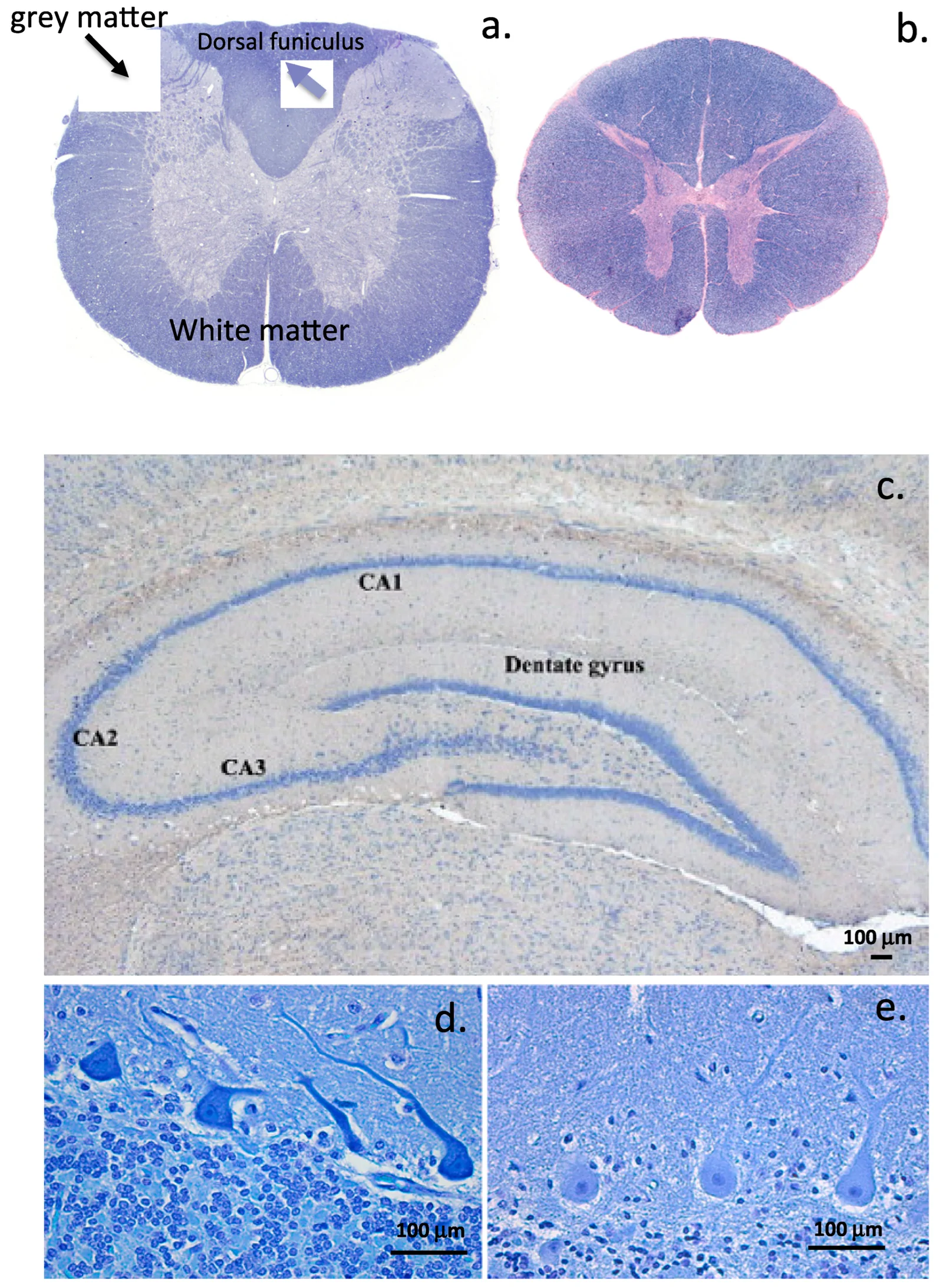
Toluidine blue staining of spinal cord and brain specimens showing the widespread distribution of GAGs. Prominent toluidine blue staining of white and grey matter is shown in low-power cross-sections of the rat (**a**) and mouse (**b**) spinal cord. Toluidine blue stains GAGs prominently in the CA1, CA2, and CA3 Cornu Ammonis regions of the hippocampal dentate gyrus (**c**). Higher-power views show toluidine blue staining of granular cells in the CA1 (**d**) and CA2 (**e**) regions. Images modified from [54,55] and accessed via Open Access.

**Figure 5:**
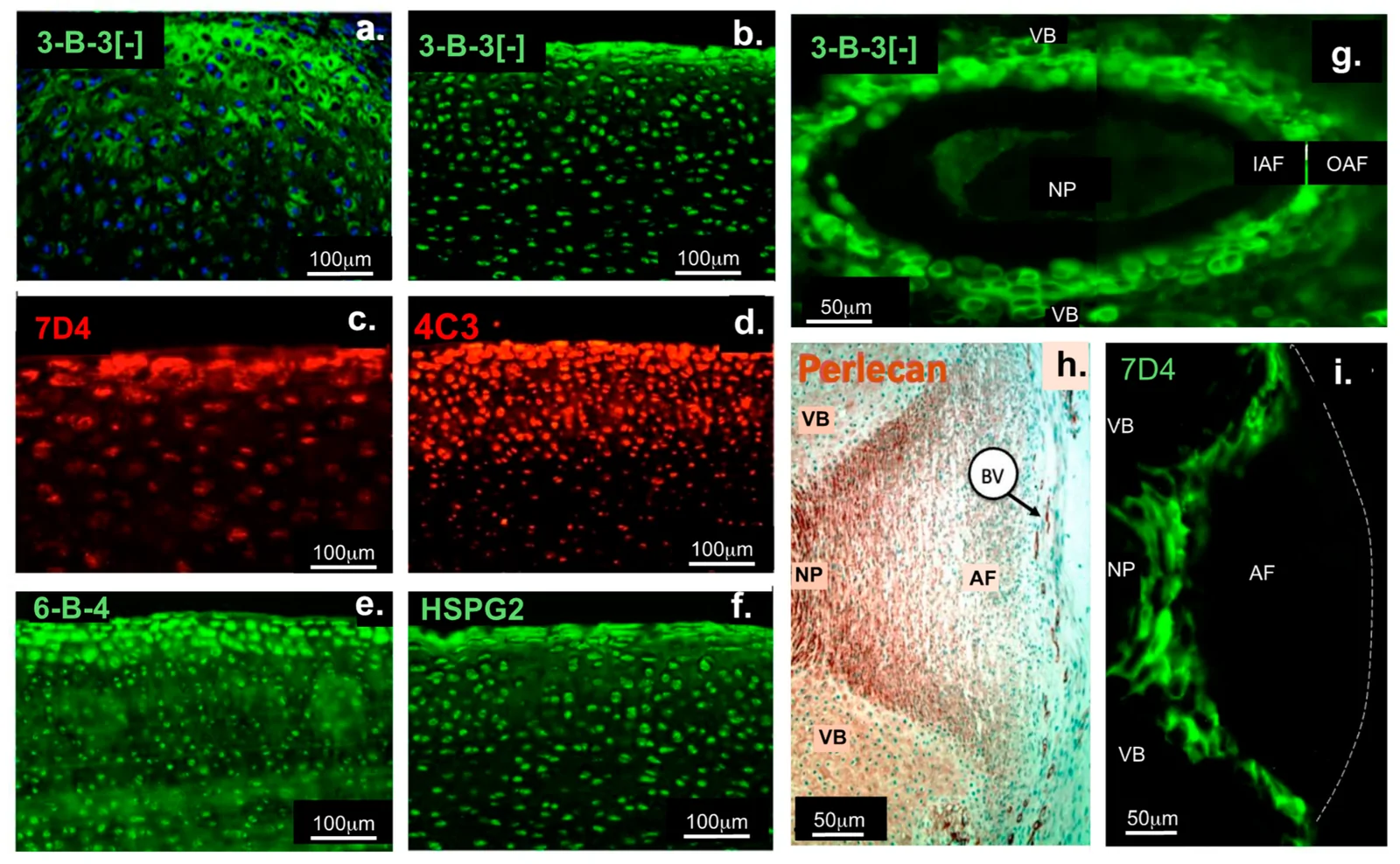
Immunolocalization of CS sulfation motifs 3B3[−], 4C3, and 7D4 in juvenile bovine cartilage and fetal intervertebral disc. Images (**a**–**f**) supplied by Dr AJ Hayes, Imaging Hub, Department of Biosciences, University of Cardiff, UK. The CS sulfation motifs detected by Mab 3-B-3[−] and 7-D-4 detect tissues undergoing morphogenetic tissue changes in development: the intervertebral disc (IVD) sections depicted are from a 14-week gestational age human specimen (**g**–**i**). The 3-B-3[−] specimen is a horizontal section (4 μm) of a bisected IVD specimen. The other two specimens are vertical sagittal sections made at 5 μm thickness. In the perlecan-stained section, small blood vessels (BVs) are evident in the outer margins of the annulus fibrosus (af) at this early stage of development. Perlecan diffusely stains the vertebral bodies, which are transient rudiment cartilages that will eventually form into bone. The inner AF/nucleus pulposus (NP) lamellar rings are evident. A more intense localization of perlecan demarcates the inner margins of the AF with the vertebral body (VB) rudiment, which is an intense region of cellular proliferation in a region undergoing morphogenetic change, which is vividly stained with the 7-D-4 CS sulfation developmental marker epitope. Cells in the inner af/np margins also stain intensely with the 3-B-3[−] CS sulfation motif. This is a region of intense metabolic activity and has been referred to as a physis in the intervertebral disc. The 3-B-3[−] epitope is also found in stem cell niches [59] and in tissue regions actively involved in growth, such as the surface region of rudiment cartilage in diarthrodial joints [120]. Images g-i modified from [121] accessed via Open Access.

**Figure 6:**
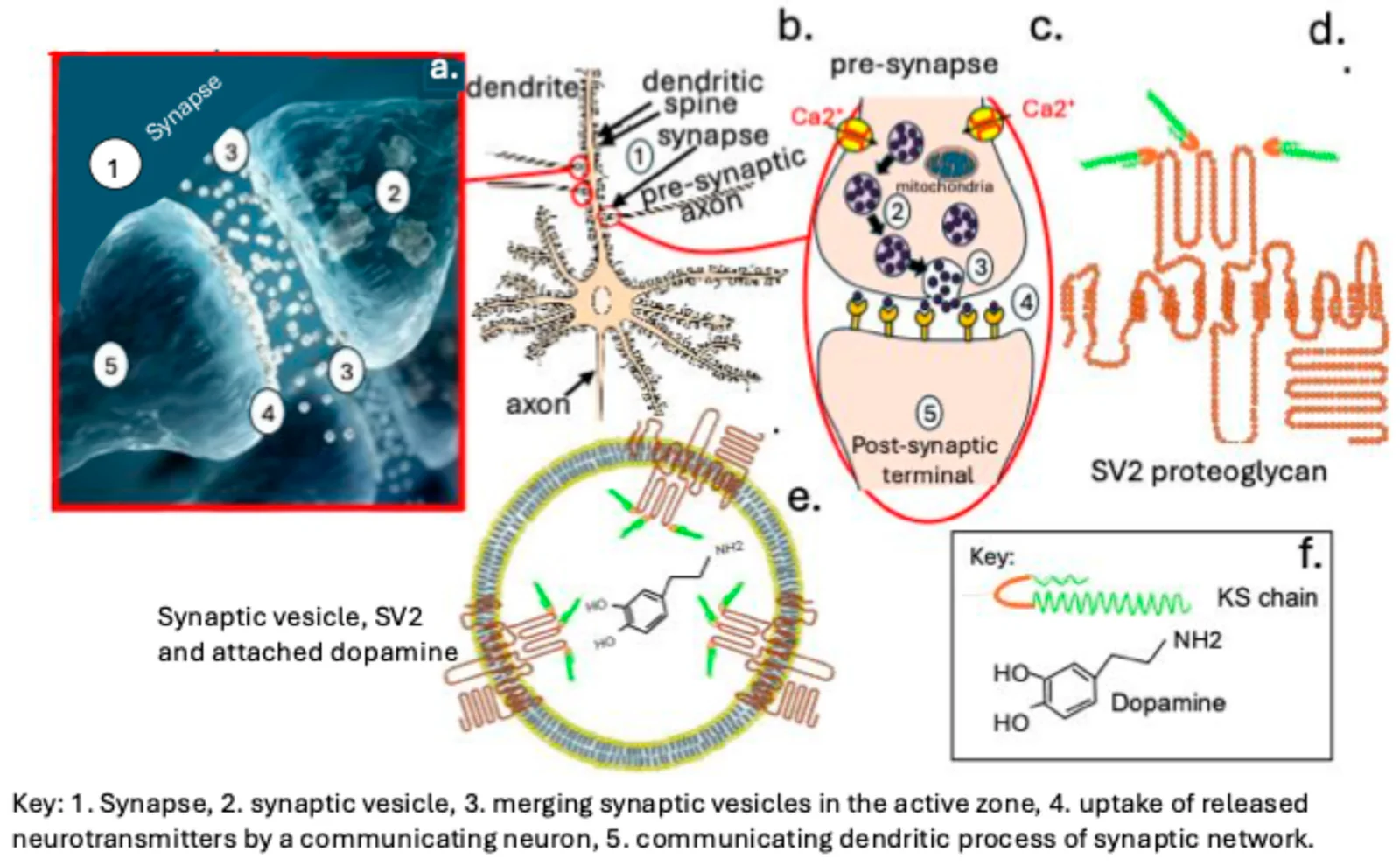
Artistic and schematic depictions of synaptic vesicle release into the synaptic gap. Panel (**a**) shows synaptic particles released into the synaptic gap, and panel (**b**) shows a neuron and its synaptic processes. Panel (**c**) provides a higher-power representation of synaptic vesicle mobilization, vesicle fusion, and neurotransmitter release after Ca^2+^ influx and depolarization of the synaptic cell membrane. Panel (**d**) shows a 12-span transmembrane KS-PG, SV2, tethered to the synaptic vesicle lipid bilayer and involved in synaptic vesicle transport. Panel (**e**) shows a synaptic vesicle with intercalated SV2 proteoglycan. The key (**f**) shows the branched KS chain and dopamine as a representative neurotransmitter transported in vesicles. Image (**a**) courtesy of the Dana Foundation.

**Figure 9:**
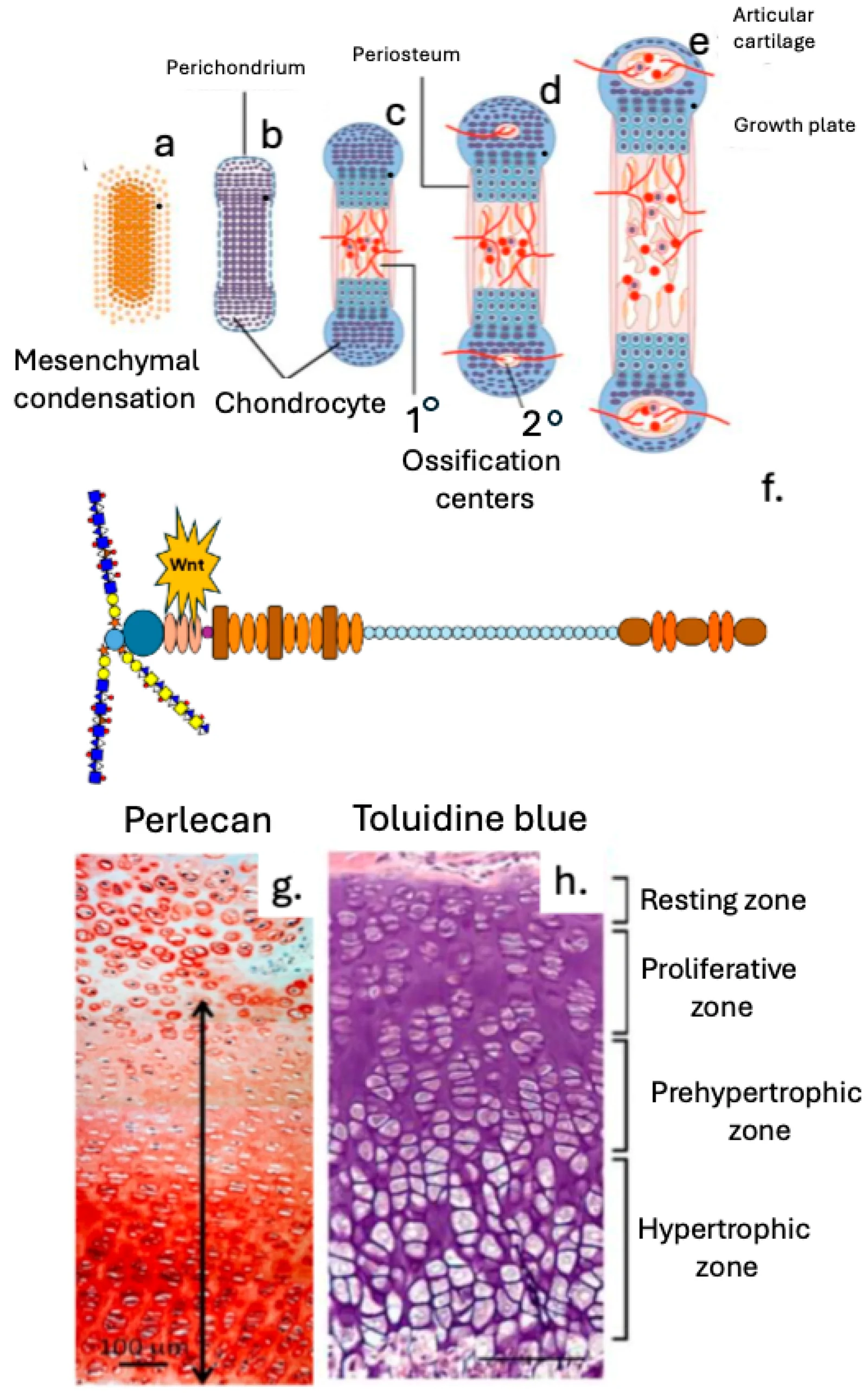
Diagrammatic depiction of the progressive stages of long bone development and endochondral bone formation in the long bones leading to extension of the axial skeleton. This is initiated by a condensation of mesenchymal cells, which develop a chondroblastic phenotype (**a**) and go through progressive maturational changes to form chondrocytes that form a perichondrium (**b**) and undergo proliferation and differentiation to extend the cartilage rudiment and form a primary (**c**) and secondary ossification center (**d**) in each pole of the developing long bone. A vascularized bone marrow cavity also forms. Further chondrocyte maturation leads to columnar formations in the cartilaginous growth plates, and terminal differentiation leads to hypertrophy and calcification of the cartilage as part of the endochondral ossification process; a vascularized marrow cavity is also now well established. The calcified cartilage also undergoes vascularization prior to calcification (**e**). Schematic depiction of the modular organization of perlecan with its 3 HS/CS chains in a domain with growth factor sequestering capability and transport. Wnt proteins bind to perlecan domain II LDLR. Perlecan acts as a carrier for Wnt proteins and IHh (**f**), and these are co-distributed in gradients in cartilage growth plates, as depicted in the immunolocalization in (**g**). A similar toluidine blue-stained growth plate more clearly depicts cell morphologies in the growth plate (**h**). Images (**g**,**h**) reproduced from [121] with permission, Open Access.

## Data Availability

No new data were created or analyzed in this study. Data sharing is not applicable to this article.
